# Eggshell Pavilion: a reinforced concrete structure fabricated using robotically 3D printed formwork

**DOI:** 10.1007/s41693-023-00090-x

**Published:** 2023-02-16

**Authors:** Joris Burger, Petrus Aejmelaeus-Lindström, Seyma Gürel, Filip Niketić, Ena Lloret-Fritschi, Robert J. Flatt, Fabio Gramazio, Matthias Kohler

**Affiliations:** 1 Institute of Technology in Architecture, ETH Zurich, Stefano-Franscini-Platz 1, 8093 Zurich, Switzerland; 2 Institute for Building Materials, ETH Zurich, Stefano-Franscini-Platz 3, 8093 Zurich, Switzerland; 3 Nicolas Fehlmann Ingénieurs Conseils SA, Place du Casino 4, 1110 Morges, Switzerland; 4 Institute for the History and Theory of Art and Architecture, Università della Svizzera italiana, Largo Bernasconi 2, 6850 Mendrisio, Switzerland

**Keywords:** 3D printing, Formwork, Digital concrete, Eggshell, Robotic fabrication

## Abstract

This paper discusses the design, fabrication, and assembly of the ‘Eggshell Pavilion’, a reinforced concrete structure fabricated using 3D printed thin shell formwork. Formworks for columns and slabs were printed from recycled plastic using a pellet extruder mounted to a robotic arm. The formworks were cast and demoulded, and the finished elements were assembled into a pavilion, showcasing the architectural potential of 3D printed formwork. The Eggshell Pavilion was designed and fabricated within the scope of a design studio at ETH Zurich. The structure was designed using a fully parametric design workflow that allowed for incorporating changes into the design until the fabrication. The pavilion consists of four columns and floor slabs. Each column and floor slab is reinforced with conventional reinforcing bars. Two different methods are used for casting the columns and floor slabs. The columns are cast using ‘Digital casting systems’, a method for the digitally controlled casting of fast-hardening concrete. Digital casting reduces the hydrostatic pressure exerted on the formwork to a minimum, thereby enabling the casting of tall structures with thin formwork. The floor slabs are cast with a commercially available concrete mix, as the pressure exerted on the formwork walls is lower than for the columns. In this research, 3D printed formwork is combined with traditional reinforcing, casting, and assembly methods, bringing the technology closer to an industrial application.

**Supplementary Information:**

The online version contains supplementary material available at 10.1007/s41693-023-00090-x.

## Introduction

The construction industry is one of the main contributors to climate change, accounting for 37% of global $\text{CO}_{2}$ emissions (United Nations Environment Programme 2021). Concrete construction is responsible for a large portion, with up to 8% of global $\text{CO}_{2}$ emissions resulting from the production of cement alone (Monteiro et al. 2017). One potential solution to make concrete construction more sustainable is to design and build material-efficient, structurally optimised structures (Favier et al. 2018; Menna et al. 2020). Several historical examples (Halpern et al. 2013; Antony et al. 2014), as well as more recent examples from research, show that it is possible to achieve material savings of 30–70% in concrete floor slabs (Meibodi et al. 2018; Ranaudo et al. 2011; Hansemann et al. 2020; Burger et al. 2022) and for structural concrete beams (Gebhard et al. 2022; Vantyghem et al. 2020; Costa et al. 2020).

However, material-efficient concrete structures often have complex, non-standard shapes, as they use material only where it is structurally needed. Non-standard shapes in concrete sharply increase construction costs, primarily due to the difficulty of producing the complex formwork needed for casting (Soto et al. 2018). Additionally, discarded formwork is currently responsible for 20–30% of construction waste (Cheng et al. 2022), a number that would potentially increase if more non-standard structures are built, as they typically result in higher formwork waste (Peurifoy and Oberlender 2011).

3D printing of formworks for concrete has the potential to provide a solution to these problems. It can increase geometrical freedom, resulting in material-efficient structures while decreasing the waste produced by the formworks. An overview of various processes used for producing 3D printed formworks is provided by Jipa and Dillenburger (2021). In particular, fused deposition modelling (FDM) 3D printing offers considerable potential as high geometric precision can be achieved at low-cost. FDM 3D Printed formwork has been used to produce a wide range of concrete elements: a concrete staircase assembled from precast individual steps (Jipa et al. 2019b), a staircase cast as a single element (Molitch-Hou 2018), a concrete canoe (Jipa et al. 2019a), concrete columns (Leschok and Dillenburger 2019; Murtha 2021), and a functionally integrated concrete floor slab (Jipa et al. 2019c).

One research project has shown that producing FDM printed formwork for a facade panel is possible at one-third of the cost of manually built wooden formwork (Han et al. 2020). Additionally, this project shows it is possible to reuse the printed formworks for multiple pours. For the facade of a high-rise apartment complex in New York, coated FDM 3D printed moulds were reused 190 times, compared to timber moulds that could be reused only ten times (Roschli et al. 2018). In case the formworks reach their end of life, it is also possible to shred them and reuse the material for a new print, as shown by Burger et al. (2023).

The present paper presents how FDM 3D printed formwork is used to cast the elements of the *Eggshell Pavilion*: a full-scale, reinforced concrete structure consisting of multiple precast elements. These are produced using the Eggshell technology (Burger et al. 2020a), a fabrication process for non-standard concrete elements that uses robotically 3D printed thin-shell formwork. The pavilion is designed and fabricated as part of the Master in Advanced Studies Architecture and Digital Fabrication (MAS DFAB) post-graduate program (Jenny et al. 2022).

The concept of Eggshell is described in detail in Sect. 2. The materials and methods used throughout this paper are described in Sect. 3. Section 4 mentions the architectural brief given to the students. Next, the resulting design process (Sect. 5) and fabrication process (Sect. 6) are described. Finally, Sect. 7 discusses the advantages and disadvantages of the design and fabrication process, whereas Sect. 8 provides the overall conclusions and an outlook to the research.

**Fig. 1:**
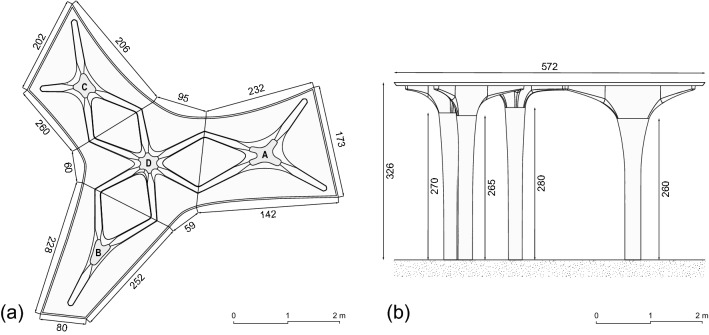
Pavilion drawings, measurements in cm. **a** Ceiling plan, **b** elevation

## Eggshell 3D printed formwork

The Eggshell fabrication process (Burger 2020a) combines robotic FDM 3D printing with the casting of fast-hardening, set-on-demand concrete (Lloret et al. 2015; Reiter et al. 2020) in a process known as digital casting (Lloret-Fritschi et al. 2020). Digital casting allows for the controlled hydration of concrete through the automated addition of admixtures during casting, enabling the use of thin, 3D printed formwork without causing breakage.

Previously, this method has been used to produce columns (Burger et al. 2020b) as well as beams (Gebhard et al. 2022). In both cases, set-on-demand concrete was essential to avoid breakage of the formwork during the casting process. However, a recent project called *RIBB3D* has explored the use of FDM 3D printed formwork combined with the casting of a conventional, self-compacting concrete to produce an optimised floor slab element (Burger et al. 2022). In those cases, it was possible to use conventional, self-compacting concrete since the floor slab was fabricated horizontally, meaning the height that has to be considered for formwork pressure was lower. Therefore, the pressure exerted on the formwork was limited.

Both of these approaches: digital casting and the casting of conventional concrete are used to fabricate the concrete elements for the Eggshell Pavilion. The pavilion consists of four columns and four floor slab elements. The columns are cast using digital casting, as it would be impossible to cast the nearly three-meter tall formworks with conventional concrete without resulting in breakage of the formwork (Burger et al. 2021). The floor slabs, however, are cast using conventional, self-compacting concrete, as this reduces complexity and cost compared to the digital casting process.

**Fig. 2:**
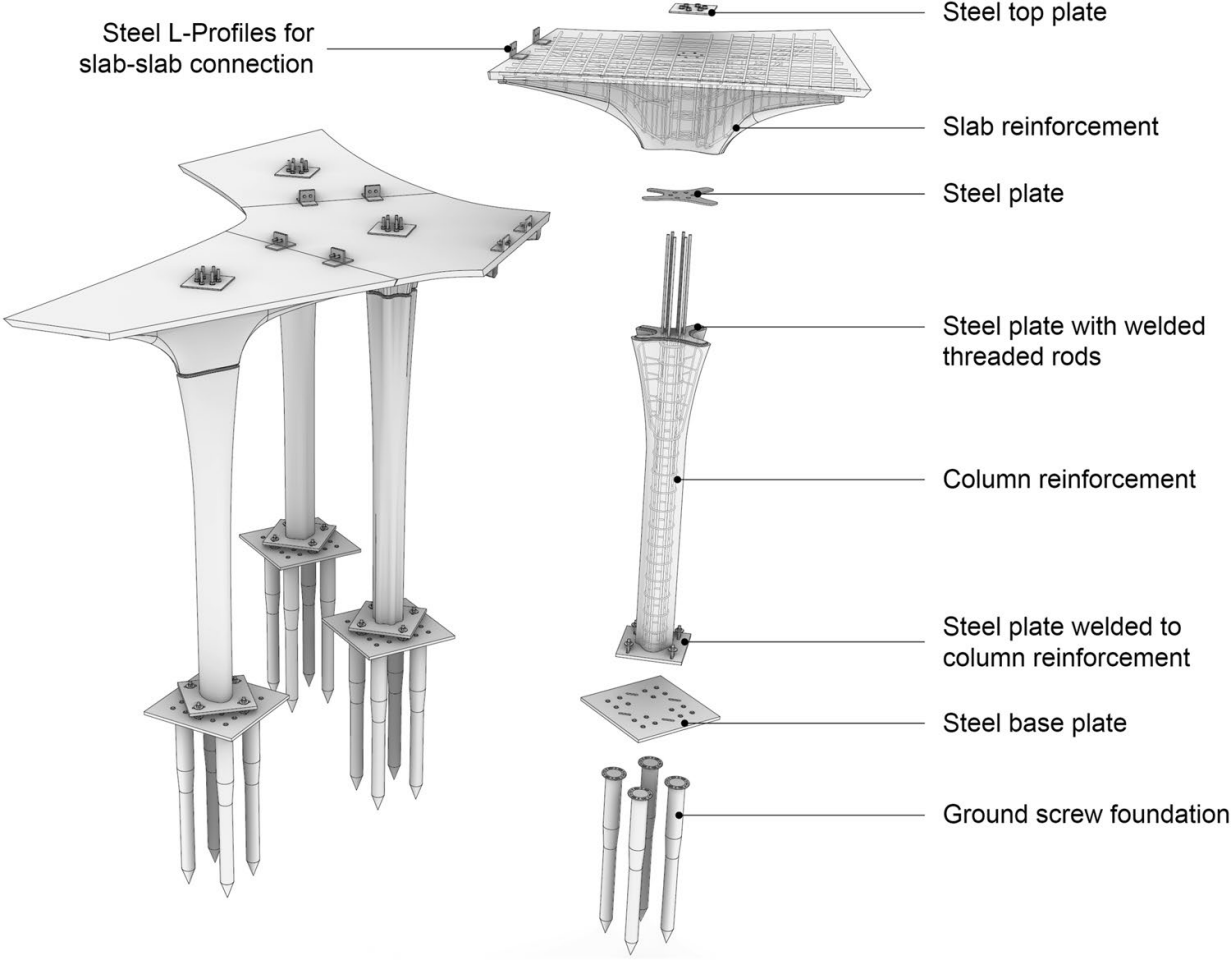
Exploded view of the pavilion, showing the connections and structural elements

## Materials and methods

### 3D printing

#### Software

The geometry for the formwork is generated using Rhino 3D and Grasshopper 3D (Robert McNeel & Associates 2020a, b). After the geometry is created, the open-source slicing package COMPAS SLICER (Mitropoulou and Burger 2020) is used to generate the toolpaths for the robotic arm. The advantage of using COMPAS SLICER is that it allows for the fast generation and precise manipulation of the toolpaths. The toolpaths are sent to the robotic arm using COMPAS RRC (Fleischmann and Casas 2020).

#### Setup

The robotic 3D printing setup is similar to the robotic setup described by Burger et al. (2022). An industrial robotic arm mounted to a ceiling gantry is used to execute the movement. The 3D printing extruder used is an E25 Pellet Extruder from the company CEAD (2021).

#### Printing materials

As printing material, PIPG from the company MCPP was used (MCPP Netherlands B.V. 2020). PIPG consists of 70% recycled polyethylene terephthalate glycol (PET-G) and 30% glass fibre (by mass). For the floor slabs (Sect. 6.2.1), the as-bought (new) printing material is used, whereas for the columns (Sect. 6.1.1), the printing material is recycled from a previous project that had involved producing a series of benches using formworks printed from the PIPG material. The formwork was removed, cleaned, shredded, and regranulated into rPIPG pellets, which were used to print the column formwork. This process is described in detail in Burger et al. (2023).

### Digital casting

For the casting of the column formworks, it is necessary to use set-on-demand casting to avoid the formwork bursting due to hydrostatic pressure from the concrete. Digital casting implements the set-on-demand casting process in a digitally controlled process. The casting uses an inline mixer into which a retarded concrete is pumped and mixed with small dosages of accelerating admixtures and superplasticisers (flow enhancers) (Lloret-Fritschi et al. 2022). Besides reducing formwork pressure, digital casting is also beneficial as it reduces the risk of environmental stress cracking under the high pH conditions imposed by concrete (Ruffray et al. 2021).

#### Setup

A detailed description of the digital casting setup is provided by Lloret-Fritschi et al. (2022). The inline mixer is a closed reactor with a volume of approximately 3 L, with double impellers rotating in opposite directions. From the bottom of the inline mixer, retarded concrete is continuously pumped using a customised conveyor pump (Type PFT Swing L). The accelerator and flow enhancers are pumped in small dosages through valves on the side of the mixer. The accelerator is processed with a ViscoTec dosing pump, while the superplasticiser is dosed with an Ismatec Reglo Digital, a peristaltic pump. After inline mixing, the material flows from the nozzle into the formwork. The flow rate can be modified between 1.5–3 L/min.

#### Casting materials

The set-on-demand concrete mix is based on the ETH Mix with 10% calcium aluminate cement (CAC) by % weight of the cement mass, as described by Lloret-Fritschi et al. (2022). The binder is composed of 56% CEM I 52.5R, 16% fly ash, and 28% limestone (by mass). The mix design includes 32% 0–4 mm sand and 8% of 4–8 mm aggregates by volume. The base concrete mix is prepared with 0.23 w/b and is retarded with the addition of sucrose to extend its open time. Additionally, the mix includes a superplasticiser to enhance the flowability.

The recipe for the accelerator paste is based on the research by Reiter (2019). It contains CAC, anhydrite, a stabilizer, and a retarder and is mixed using the method described by Boscaro et al. (2022). The accelerated concrete (retarded concrete and accelerator) has a 28-day average cube compressive strength of 82 MPa.

### Conventional casting

The concrete used for casting the floor slabs was a self-compacting mix with a maximum aggregate size of 4 mm. The reason for the small aggregate size was to ensure the fluidity of the mix. The mix was designed as concrete class C40/50, had an average cube compressive strength of 63.4 MPa at 28 days, and used circular Susteno 4R cement.

### 3D scanning

A Leica RTC360 3D Laser Scanner (Leica Geosystems 2022) set to fine resolution (accuracy of 1.9 mm below 10 m) was used for 3D scanning of the finished elements. In each case, the four concrete elements (columns and floor slabs) were positioned in a square, and nine scans were taken, eight on the outside of the square and one on the inside, amounting to four complete scans per element. These scans were linked together using the Leica Cyclone REGISTER 360 software (Leica Geosystems 2022). The resulting point clouds were then exported for further processing. The open-source point cloud processing software CloudCompare (2022) was used to clean the point cloud of any points not relevant to the scanned element. Additionally, the resulting point cloud was subsampled using a minimum distance per point of 3 mm to ensure an even point density. Then, the scanned point cloud was compared to the geometry used for 3D printing the formwork, using the workflow outlined in CloudCompare (2015).

## Design studio and design brief

The design and fabrication of the pavilion were realised within the scope of the MAS DFAB at ETH Zurich. During the second trimester, the Integrated Design and Robotic Fabrication Project (Jenny et al. 2022), the architectural potential of an existing research project is explored, in this case, the Eggshell research. The students were tasked to design and fabricate a pavilion for a six-month exhibition at the Vitra Design Campus in Weil am Rhein, Germany, after which the pavilion was dismantled and transported to a permanent location. To be able to conceptualise, design, and fabricate the pavilion within a limited time of ten weeks, it was necessary to provide a studio brief with precise guidelines in terms of design, dimensions, fabrication, transport, and assembly.

Additionally, the pavilion must be prefabricated using the existing fabrication setup (Sect. 3), the elements must fit within standard lorry dimensions, be assembled using dry joints, comply with building regulations (Sect. 5.2), and both the design and fabrication of the elements have to be conducted within ten weeks. Lastly, the need of integrating steel reinforcement and providing adequate concrete cover of the reinforcement are key constraints. As it is challenging and expensive to bend reinforcement bars in two directions (double curvature), the reinforcement bars are limited to bars bent in one direction (single curvature). These aspects can be summarised in the following requirements:

- A canopy of around ${15}~{\text{m}}^{2}$.
- The pavilion should have a minimum of 6 and a maximum of 10 column- and slab-like elements.
- The bounding box of the elements on its shortest dimension must be less than 2.4 m and in the longest dimension 2.8 m.
- The columns can be up to 3 m tall.
- Total printing time must be less than 120 h.
- Maximum volume of ${3}{\text{m}}^{3}$ of concrete.
- Reinforced with single curved reinforcement.
- Minimum concrete cover of 20 mm, maximum concrete cover 60 mm.
- Possible to assemble using one crane and two people.

The project was developed in three phases. (1) During the initial phase (two weeks), the students explored six projects in parallel that were then combined into one design concept. (2) In the second phase (4 weeks), the students were organised into five groups focusing on five topics: the overall design, the design of the surface texture, the formwork design and fabrication, the design of reinforcement and connections, and the robotic 3D printing. A combined parametric model was developed that each group contributed to in parallel. (3) In the third phase (4 weeks), the elements were fabricated.

## Design

The pavilion was designed as a non-orthogonal column-slab system with non-standard elements (Fig. 1). It consisted of four columns with four slab-like elements organised as column-slab pairs. Each pair was self-stabilising, with the centre of mass of the slab located on top of each column. The overall height of the pavilion was 3.26 m, with the tallest columns being 2.8 m. The maximal span was 2.15 m, and the longest cantilever was 1.52 m. The thickness of the slab was 6 cm, and the minimal cross-section of the ribs was $6\times 18$ cm (without the slab). One column-slab pair was placed in the centre, while the other three were configured around the central pair. The columns were designed to emphasise the ability of the fabrication method to produce complex geometry with a high surface resolution. The slabs were designed as a rib structure that supports a 6 cm thick concrete slab. The rib structure on the slabs continued on the top of the columns and gradually translated into a vertical texture inspired by the ribs. The detailing between columns and slabs (Fig. 2) was purposely left visible to create a visual separation between the columns and slabs, thus softening the colour difference between the different elements.

### Computational design workflow

**Fig. 3:**
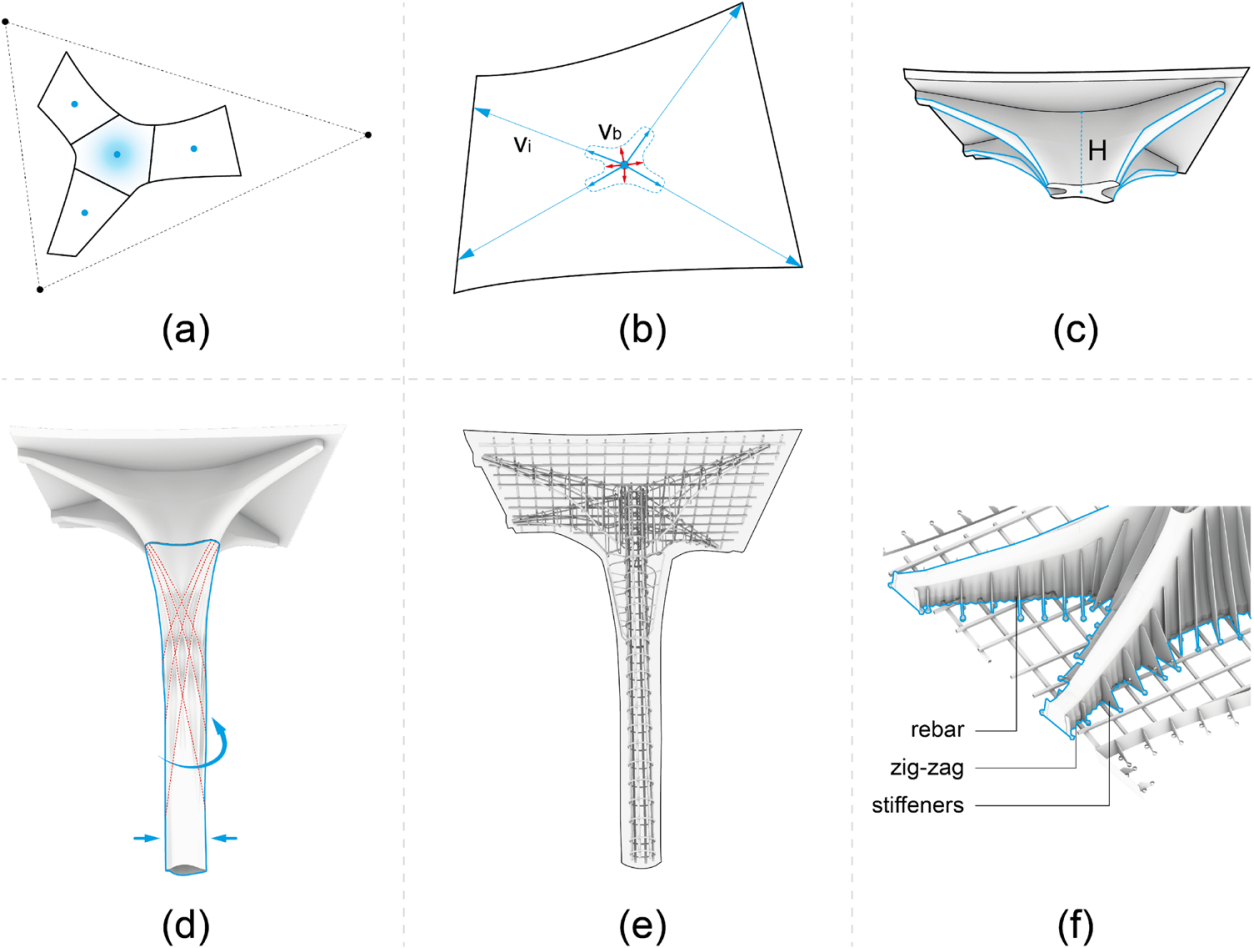
The computational workflow. **a** Define the pavilion outline and divide it into four elements. **b** Generate initial vectors $(V_{\textrm{i}})$ for the ribs, bisecting vectors $(V_{\textrm{b}})$ for the columns, and generate column top profile. **c** The geometry of the capital is generated, with the height (*H*) based on the area of the slab. **d** The geometry of the column is generated by scaling and rotating the cross-section. The pattern is generated based on the geometry. **e** Generation of reinforcement. **f** Generate final formwork geometry, including holes for the reinforcing bars, zig-zag pattern, and formwork stiffeners

The computational design model was based on Rhino 3D and Grasshopper 3D. The input for the parametric model was an abstraction of the intended outline of the roof represented by a triangle specified by three points. The designer could modify the orientation and geometry of the pavilion by moving the points. This first step of the parametric model returns the outline of the pavilion, after which it is divided into four elements, and the centre of gravity of each element is calculated (Fig. 3a).

The ribs were generated from initial vectors $({V}_{\textrm{i}})$ defined from the centre of gravity to the corner of the elements. If the angle between two vectors is smaller than $50^\circ$, the two vectors are replaced by the mean vector. From these initial vectors, bisecting vectors $({V}_{\textrm{b}})$ were calculated. The two sets of vectors are then combined to create a spline profile which defined the shape of the contact surface between the slabs and columns (Fig. 3b). Then, the height of the capital was determined based on the area of the slab. The geometry of the capital was defined by section profiles along the initial set of vectors that were lofted together to form the capital (Fig. 3c).

The columns were generated by copying, translating, simplifying, rotating and scaling the spline profile towards the base of the column, from which a loft surface was generated. The column surface texture was computationally generated based on the geometry of the column. However, the designer was able to intervene manually and create different texture variations (Fig. 3d).

From these parametric surfaces (four slabs and four columns), the reinforcement and formwork geometry was generated. The reinforcement was generated using a parametric model that incorporated the structural requirements (such as minimum concrete cover distance) and fabrication requirements (such as minimum bending radius of the reinforcement bars). The parametric model also produced the necessary drawings to fabricate the reinforcement bars and cages (Fig. 3e).

The final step of the computational tool was to prepare the geometry of the formworks for printing. For the floor slabs, stiffening ribs and holes for the reinforcement bars were added (Fig. 3f). For both columns and floor slabs, fixing features were added, allowing the formwork to be screwed into the printing platform (Burger et al. 2022).

**Fig. 4:**
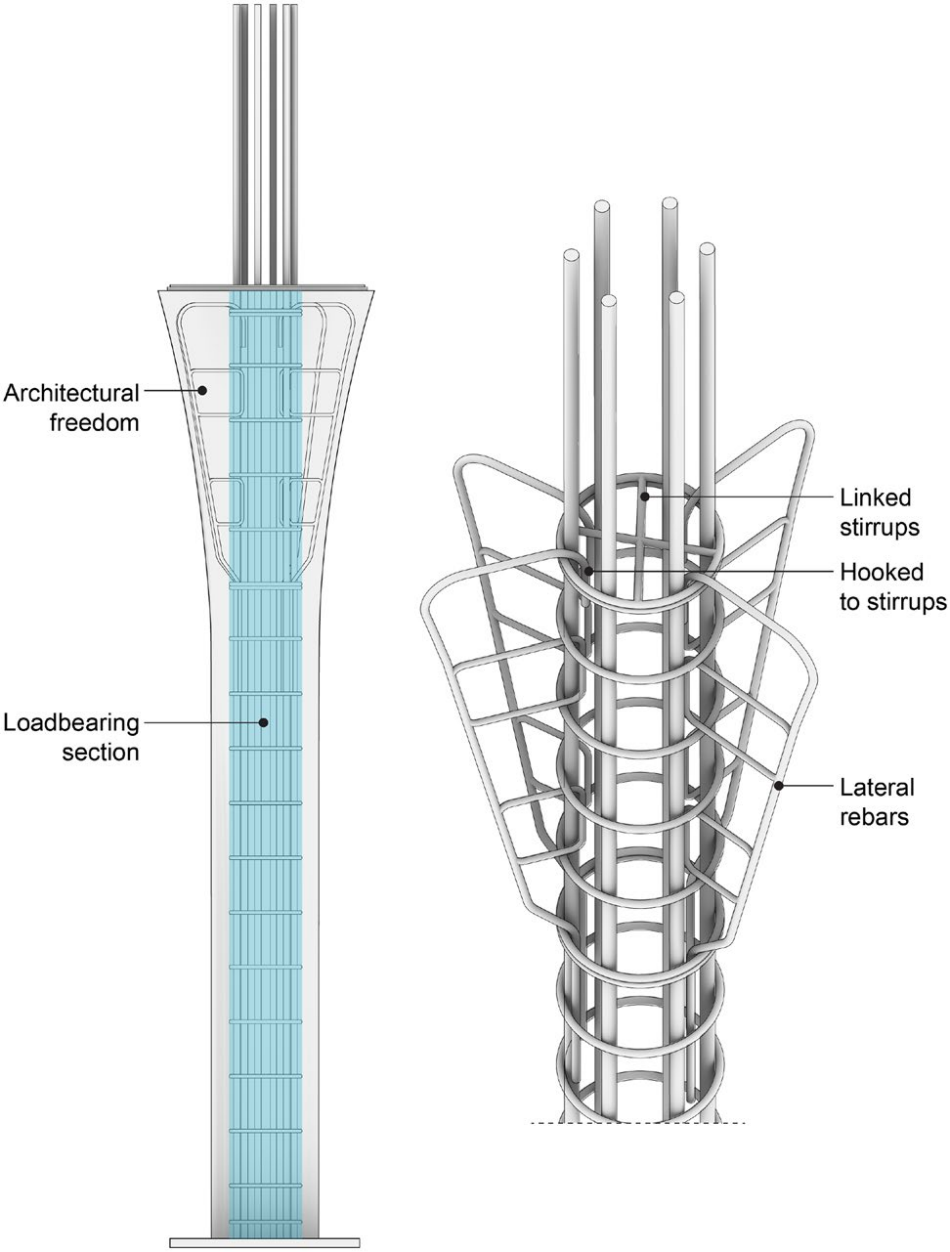
Column reinforcement. The blue area indicates the load-bearing section of the column

### Structural design

The structural design principles of the Eggshell Pavilion aimed at providing architectural freedom whilst being tailored to the unique fabrication process and satisfying the structural requirements. The following section describes some of the constraints leading to the choice of static system and structural detailing.

As mentioned in Sect. 4, a set of constraints was established to guide the design process. From a structural perspective, the most important constraints were: (1) maximising design freedom while ensuring a reliable structural concept, (2) staying within the given budget for material use, and (3) reversible connections between the elements.

A structural system consisting of simple yet efficient load-carrying mechanisms was used (see Fig. 2). The column-slab pairs were joined using a dry mechanical connection designed to take axial and shear forces as well as bending moments. Four micro-piles, in the form of ground screws, were used as the foundation system, allowing the efficient transfer of the internal forces from the structure to the soil. Each of the four column-slab pairs was auto-stable, meaning they were designed as independent isostatic systems. The columns were placed as close as possible to the centre of gravity of the ribbed slab, which resulted in multiple benefits:

1. Reduced bending moments allowing for slender columns.
2. An ultra-thin reinforced concrete slab.
3. No transfer of forces between slabs, allowing for a simplified slab-slab connection.
4. A simple assembly procedure.

#### Columns

The design process (Sect. 5.1) resulted in each of the four columns having a unique cross-section, starting with a 20 cm diameter circle at the bottom and finishing with a star-shaped cross-section on the top. The columns carried similar internal forces; thus, the reinforcement layout was nearly identical for all four columns (Fig. 4) and consisted of six longitudinal bars Ø18 with Ø10 stirrups placed every 10 cm. Although each column had the same general reinforcement layout, the exact spacing and length were different due to the differences in diameter and height. The centre part of the column was load-bearing, whereas the variation in the outer part resulted from design expression.

Additionally, the top part of each column was reinforced with lateral reinforcing bars (Fig. 4) to guarantee the behaviour of the elements under the Serviceability Limit State (SLS). Deviating the compression stresses in the capital required transversal reinforcing bars; therefore, lateral reinforcing bars were hooked to a linked stirrup, which prevented them from deforming under tension (Fig. 4). On the bottom, the longitudinal reinforcing bars were welded to a steel base plate to transmit the internal forces to the ground screws.

#### Floor slabs

The main challenge in the structural design of the ribbed reinforced concrete slabs was the thin element, which extends beyond the values typically recommended by the design norms [i.e. SIA 262 (sia: Swiss Society of Engineers and Architects: Normen, n.d.), EC (European Commission, n.d.), ACI (American Concrete Institute, n.d.)]. As the slab is only 6 cm thin, the tensile strength at the Ultimate Limit State (ULS) must be considered. Since this parameter varies and is difficult to predict, the cracking resistance of the 6 cm slab was calculated using the tensile concrete strength corresponding to the 5% fractal on the standard bell curve (70% of the mean value). A partial safety factor for concrete (equal to 1.50) was applied to ensure that the slab never passes its cracking limit. The ribs were distributed according to these values to ensure that the tensile strength of the concrete was not surpassed.

For the 6 cm thin solid slab, galvanised steel reinforcement bars Ø12 were placed every 15 cm of the thin slab. Galvanised steel was chosen over conventional steel due to the small concrete cover (20 mm). The ribs were reinforced with prefabricated cages placed inside the printed formwork. The continuity of the top reinforcement was guaranteed by multiple overlapping reinforcement bars Ø12.

As the ribbed slabs were cast upside down, they needed to be flipped before assembly, resulting in a risk of positive bending moments in the ribs. Therefore, the bottom reinforcing bars were hooked on linked stirrups (similar to the top of the columns as described in Sect. 5.2.1).

**Fig. 5:**
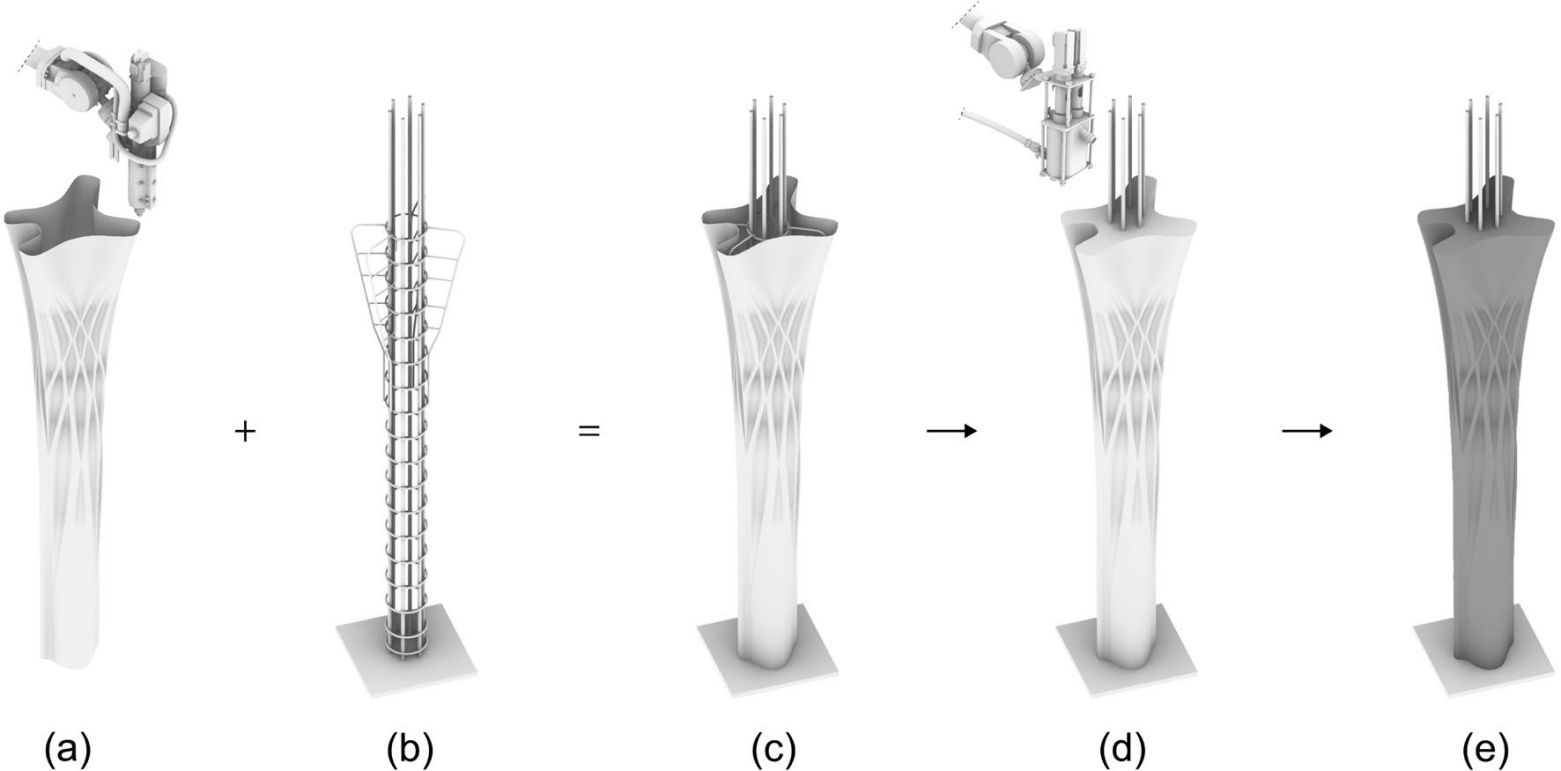
Fabrication sequence of the columns. **a** 3D Printing of the formwork, **b** prefabricated reinforcement, **c** place formwork around reinforcement cage, **d** digital casting, **e** demoulding

#### Foundation

Micro-piles in the form of ground screws were used to create a stable yet temporary foundation. Four KRINNER screws type KSF M 76x100-M12 (KRINNER, n.d.) with 40 cm distance were placed beneath column-slab pair (see Fig. 2). This solution ensured the efficient transmission of bending moments and normal forces and allowed for simple and reversible assembly and disassembly of the structure.

#### Connections

Three types of connections, inspired by solutions typically applied in steel structures, were used to assemble the pavilion: (1) column-to-ground screws connection, (2) column-to-slab connection, (3) slab-to-slab connection (Fig. 2).

The column-to-ground screw connection consisted of two steel plates and four bolts. One of the steel plates was directly welded to the reinforcement cage of the columns (as described above), and the other was bolted on top of the ground screws. Structurally, the connection is considered completely rigid and thus able to transmit the bending moments as well as shear and normal forces from the column to the foundation.

The column-to-slab connection consisted of two steel plates placed between the column and the slab to avoid concrete-to-concrete contact and local crushing of the elements. Six threaded bars, welded to the reinforcement cage of the columns (as described in the section above), passed through the ribbed slab and were joined on top with another steel plate to ensure a rigid connection between the column and the slab.

Finally, the slab-to-slab connection consisted of two L-shaped profiles that were glued with epoxy resin on the slabs and then bolted together. This detail was a constructive connection used during assembly to correctly align the elements and keep equal spacing between the four segments of the pavilion.

## Fabrication

A video documenting the fabrication process can be viewed here: https://vimeo.com/752965274.

### Columns

**Fig. 6:**
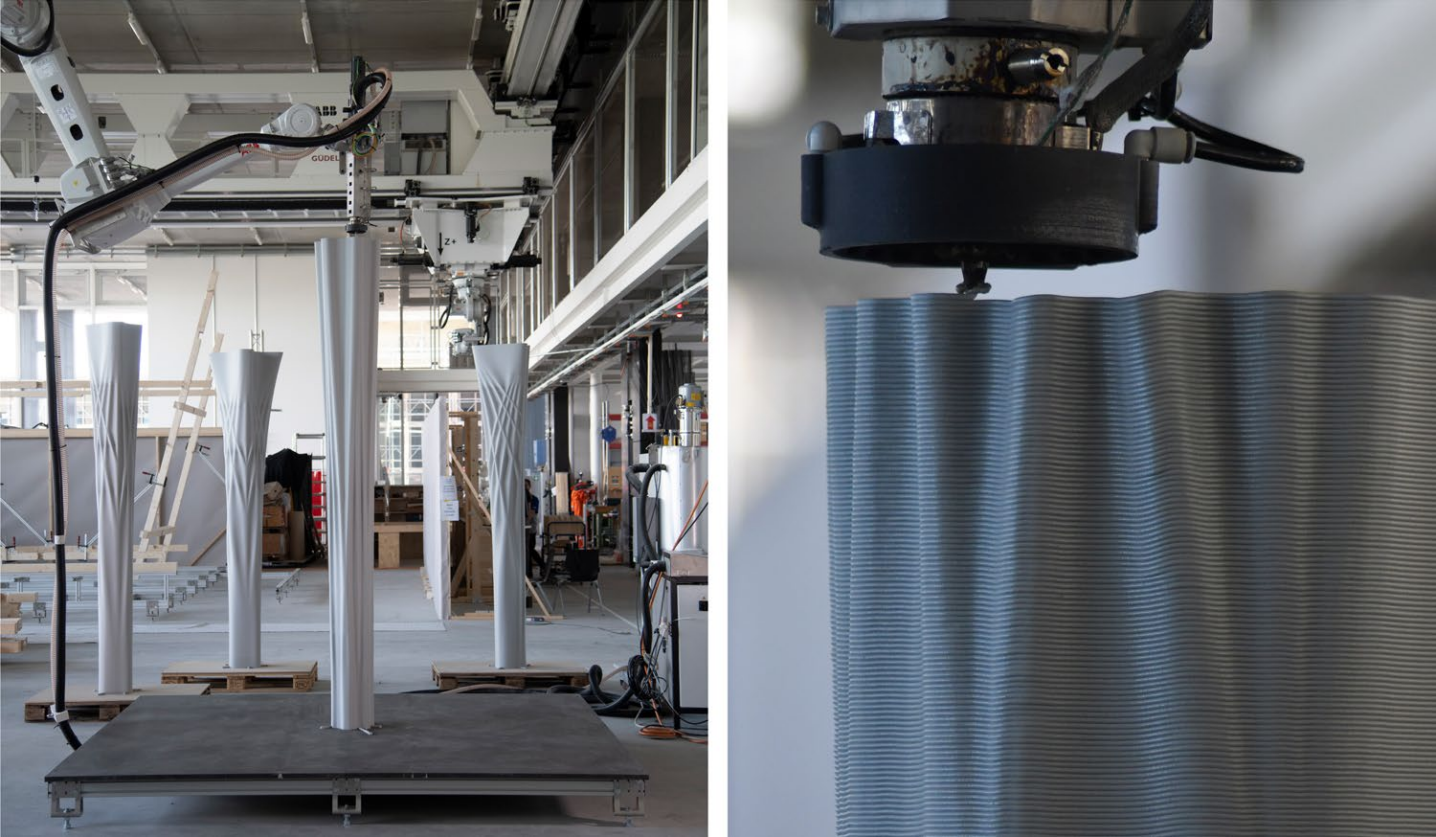
3D Printing of the column formworks

The four columns were fabricated using the Eggshell consecutive fabrication process introduced by Burger et al. (2020b), combined with the digital casting process described by Lloret-Fritschi et al. (2022). The process (Fig. 5) consists of the following steps: (a) 3D Printing of the formwork, (b) prefabricating reinforcement, (c) combining formwork with reinforcement, (d) digital casting, and (e) demoulding.

**Table 1:** Fabrication parameters of the columns

	Column A	Column B	Column C	Column D
Printing time	7 h 11 m	8 h 7 m	6 h 40 m	7 h 50 m
Toolpath length (m)	1507	1260	1339	1437
Casting time (min)	150	110	115	112
Volume (L)	210	158	165	163
Height (mm)	2600	2650	2700	2800

#### 3D printing

The columns were 3D printed using the fabrication setup described in Sect. 3.1.3. The recycled PIPG material was used for printing. A nozzle with a diameter of 3 mm was used, a layer height of 2 mm, and a layer width of 3 mm.

The printing speed was adjusted based on the toolpath length of each printing layer. Experiments have shown that with the parameters mentioned above, a minimum time per layer of 15 s is required to allow the layer to cool sufficiently before the next layer is printed.

The column formworks were printed in around 6–8 h (Table 1, Fig. 6). The large difference in printing time can be explained by the fact that some prints had interruptions during the process.

As the column height exceeded the range of the robotic arm, it was necessary to move the gantry vertically. For Column A, the vertical axis was moved up by 2 mm (the layer height) in every layer. This movement, however, resulted in a visible seam between each layer. For that reason, the process was adapted so that the gantry moved up with every point of the printpath. For example, if the toolpath of a layer has 100 points, the robotic arm would move up by $2\,\text{mm}/100=0.02\,\text{mm}$ for every point, which resulted in a much smoother seam between layers.

**Fig. 7:**
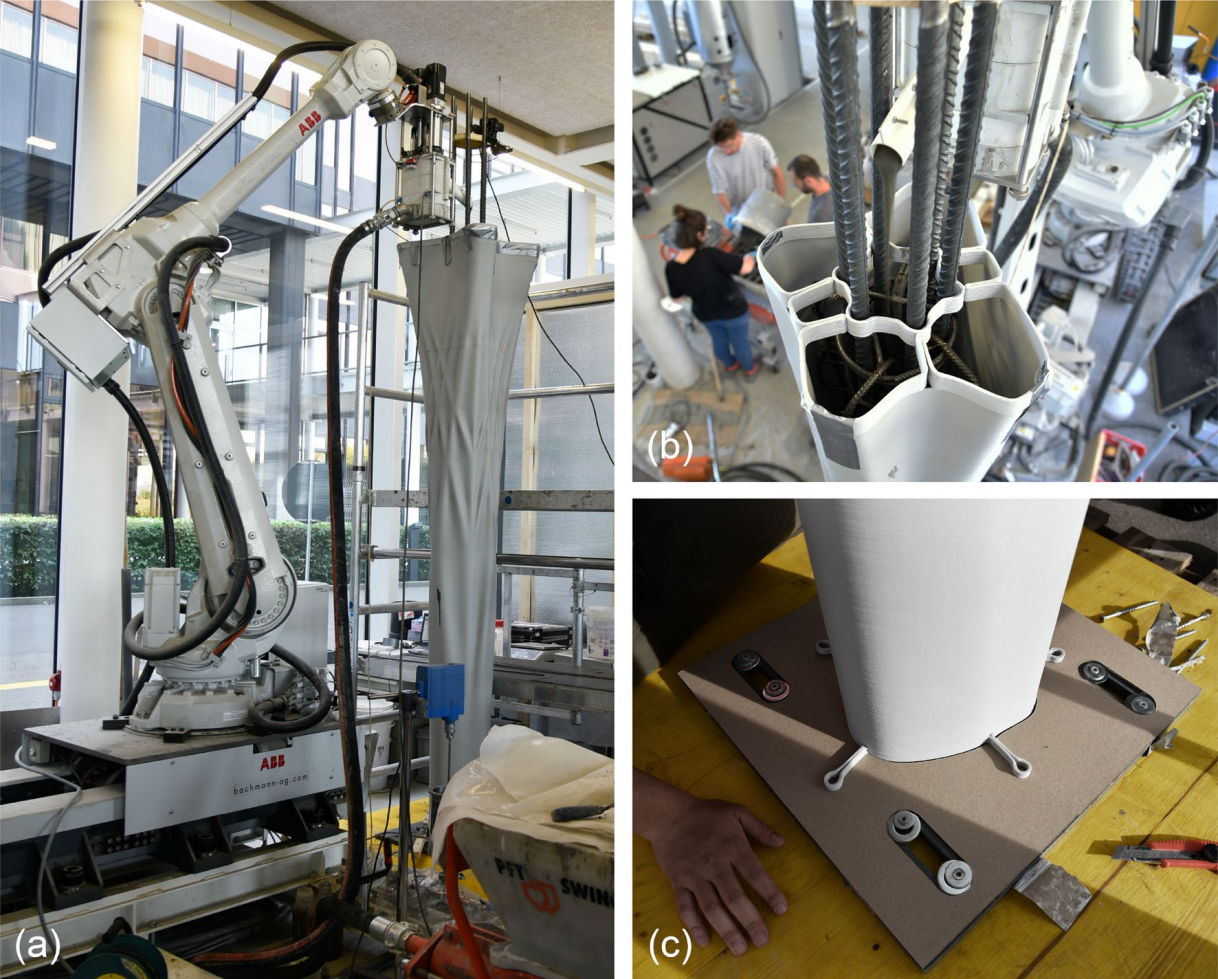
**a** Digital casting process of a column, **b** 3D printed top template, **c** laser-cut bottom template

**Fig. 8:**
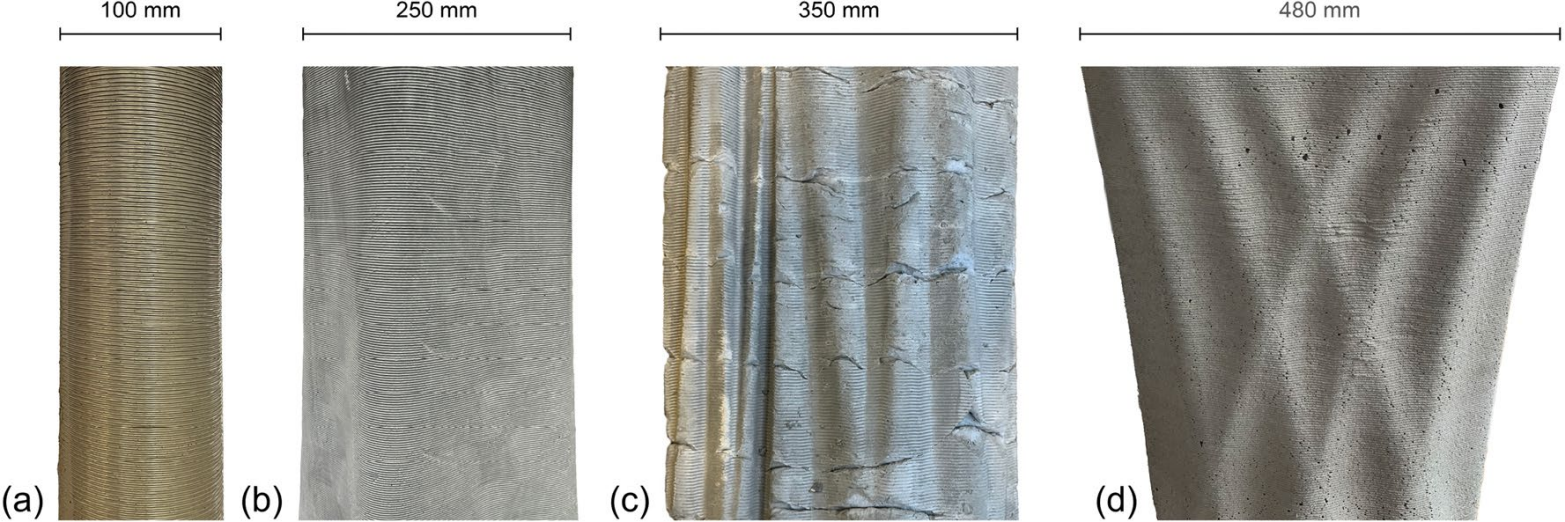
Surface close-up of column prototypes. **a** First prototype $d = 100$ mm, **b** second prototype $d = 250$ mm, **c** third prototype $d = 350$ mm, **d** fourth prototype, top $d = 480$

#### Reinforcement

The reinforcement of the columns was prepared as prefabricated reinforcement cages with additional reinforcing bars. The primary cylindrical reinforcement cage was prefabricated in one piece, whereas the additional bars for the column ribs were added separately. As the reinforcement cage was welded to the foundation plate (Sect. 5.2.1), the formwork had to be placed around the reinforcement from the top. However, as the top of the column was wider than its base, the reinforcement cage could not be fully assembled before placing the formwork. Therefore, the additional bars for the ribs were added after the formwork had been placed around the reinforcement. The reinforcement and formwork were aligned using 3D printed templates at the top of the column (Fig. 7b) and laser-cut templates at the bottom (Fig. 7c).

#### Casting

The columns were cast using the digital casting setup described in Sect. 3.2.1 (Fig. 7a). A batch of retarded concrete is mixed, and the concrete, accelerator, and superplasticiser are pumped into the inline mixing reactor from the bottom. The accelerated concrete exits from the top outlet and flows into the column formwork with a continuous flow rate of 1.5 L/min. Although the system was previously operated with a maximum flow rate of 3 L/min (Lloret-Fritschi et al. 2022), this had not been extensively tested; therefore, the flow rate was reduced to half to be on the safe side. Casting times for the columns ranged between 110–150 min, depending on the volume of each column (Table 1).

Before casting the final four columns, several prototypes were cast. First, a cylinder with a diameter of 100 mm and height of 1500 mm was cast, resulting in good surface quality (Fig. 8a). Then, a non-standard column with a height of 2800 mm and an average diameter of around 250 mm was cast. This second prototype also showed good surface quality (Fig. 8b). The third prototype aimed to represent the casting of a column with similar geometry to the final columns, with an average diameter of around 350 mm. This prototype showed surface defects, with visible gaps in the concrete approximately every 50 mm (Fig. 8c). Most likely, the concrete was not fluid enough to reach the edges of the larger diameter formwork. As the concrete is hardening quickly due to the addition of the accelerator, it stiffens before flowing to the formwork edge.

Two strategies were implemented in an attempt to improve surface quality. (1) Previously, the retarded concrete was mixed to have a slump spread flow of 600 mm right after mixing. By increasing the amount of superplasticiser added to the retarded mix, the slump spread flow was increased to 670 mm. (2) Additionally, the column formwork was vibrated using a vibration needle on the outside of the formwork. Although vibrating the concrete inside the formwork would have been more effective, this was impossible due to limited access inside the tall formwork. These two strategies helped improve the surface quality of a fourth prototype (Fig. 8d).

**Fig. 9:**
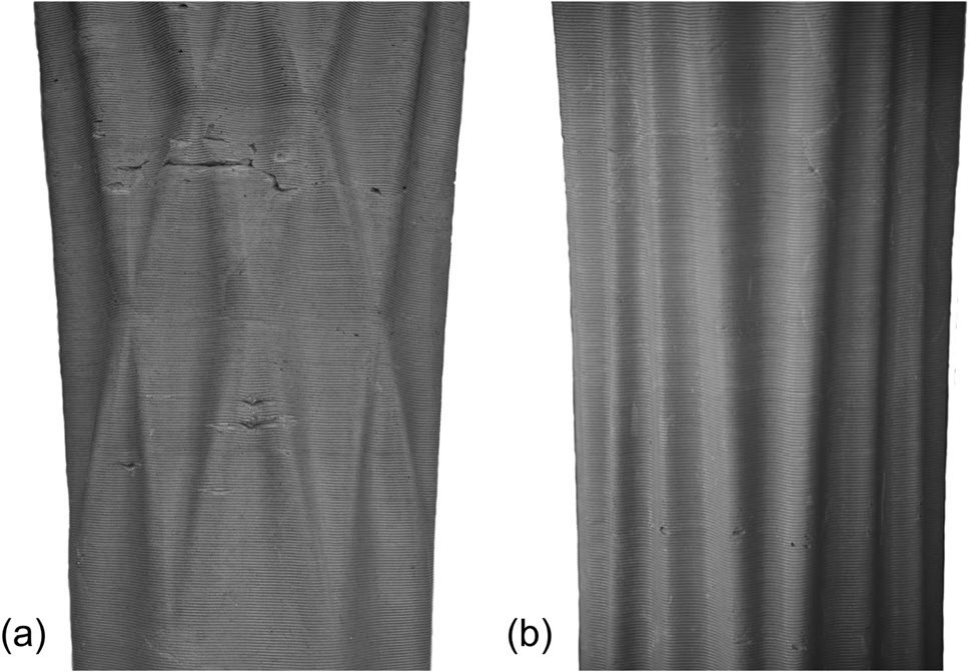
Surface close-up of two of the columns. **a** Column B, **b** column D

The four columns were successfully cast using the digital casting setup. However, some surface defects can still be identified, which indicates that the implemented solutions (increasing spread flow and formwork vibration) did not fully solve the issue (Fig. 9). Additionally, problems occurred during the casting of Column A. After 120 min of casting, accelerated concreted started hardening inside of the reactor, resulting in clogging that caused the impellers to stop rotating. Therefore, it was impossible to continue cast using the inline mixer, and the last 20 L of concrete had to be cast and accelerated manually, using the method described in Burger et al. (2020b). The cause of clogging is not yet fully understood, but possible explanations might be a temperature increase in the inline mixer and a contribution from the high ambient temperature.

**Fig. 10:**
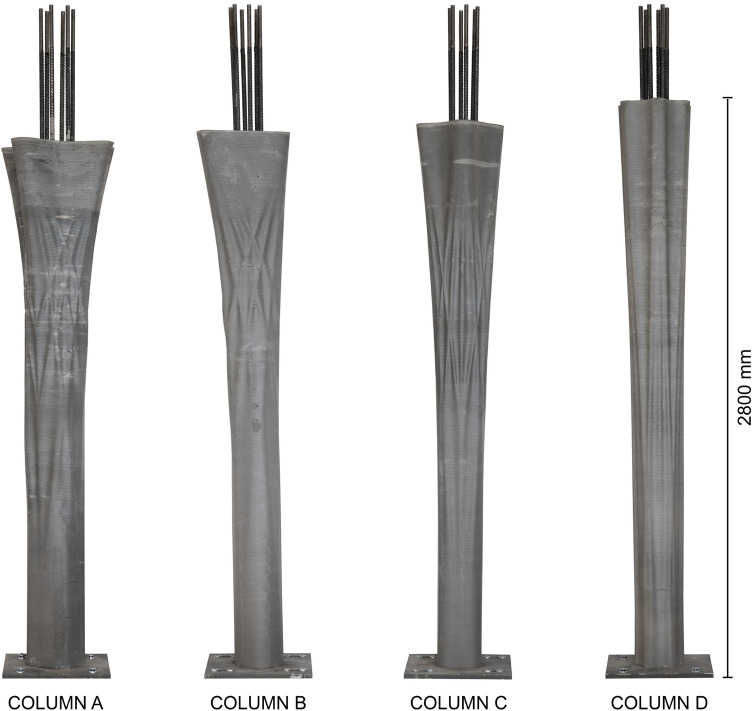
The four completed columns

The columns were demoulded 5 days after casting (Fig. 10). Demoulding was done by heating the plastic formwork locally using a hot air gun and making a vertical cut in the formwork. Once the formwork has been cut from top to bottom, it can be removed from the column. Cutting the formwork has to be done carefully to avoid scratching the surface of the concrete.

**Fig. 11:**
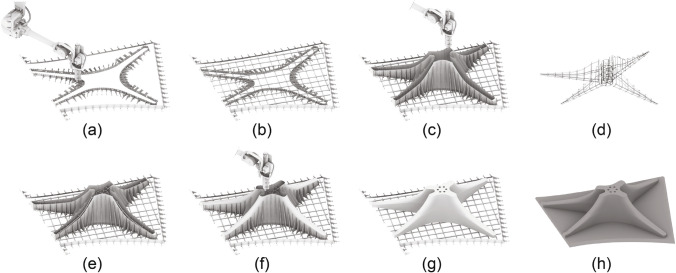
Fabrication sequence of the floor slab elements. **a** 3D Printing of the slab boundary, **b** placing slab reinforcement, **c** 3D printing the capital, **d** prefabricated reinforcement cages, **e** insert reinforcement, **f** 3D printing the cap of the capital, **g** casting of the capital, **h** casting of the solid slab section

### Floor slabs

The four floor slab elements were produced using a similar fabrication process as in the RIBB3D project (Burger et al. 2022). The fabrication process consists of the following steps (Fig. 11):

- 3D Printing of the slab boundary and the first 20 mm of the capital walls.
- Placing of the solid slab reinforcement.
- 3D Printing of the formwork walls.
- Prefabricating the reinforcement.
- Placement of the capital reinforcement.
- 3D Printing the cap of the capital.
- Casting the capital.
- Casting the solid slab section.

The slabs were fabricated on top of a custom-built platform made from plywood supported by wooden beams. Each slab had its individual platform so that the platform could be used during every step of the process. First, the formwork was 3D printed on top of the platform and fastened using screws. Then, the entire 3D printed formwork, including reinforcement, could be moved to the casting location, where the formwork was cast. After demoulding, the finished slab was connected to the platform again to be used for transport. Finally, the platform was used for the flipping process of the slabs, as they had been fabricated upside down and had to be flipped 180$^\circ$ before assembly.

**Table 2:** Fabrication parameters of the floor slabs

	Slab A	Slab B	Slab C	Slab D
Printing time				
Boundary	1 h 31 m	1 h 25 m	1 h 28 m	1 h 10 m
Capital walls	8 h 45 m	8 h 22 m	8 h 4 m	7 h 57 m
Capital caps	8 h 29 m	7 h 31 m	9 h 44 m	8 h 7 m
Total	18 h 45 m	17 h 18 m	19 h 16 m	17 h 14 m
Volume (L)				
Capital	352	194	212	213
Slab	170	132	120	154
Total	522	326	312	367
Height (mm)	660	610	560	460

#### 3D printing

The formworks for the floor slab elements were 3D printed using the same equipment and setup as the column formworks. In this case, the as-bought (new) printing material was used, as there was not enough recycled material available to print the slab formworks. Relevant printing parameters were: a nozzle diameter of 6 mm, layer height of 3 mm, and layer width of 5 mm. Compared to the column formworks, the layer height was increased to speed up the printing process, and the layer width was increased to create a stronger formwork. Due to the increased layer height and width, a nozzle with a larger diameter was used.

The 3D printing processes consisted of three parts: (1) the outer boundary (Fig. 11a), (2) the formwork walls (Fig. 11c), and (3) the cap (Fig. 11f).

**Fig. 12:**
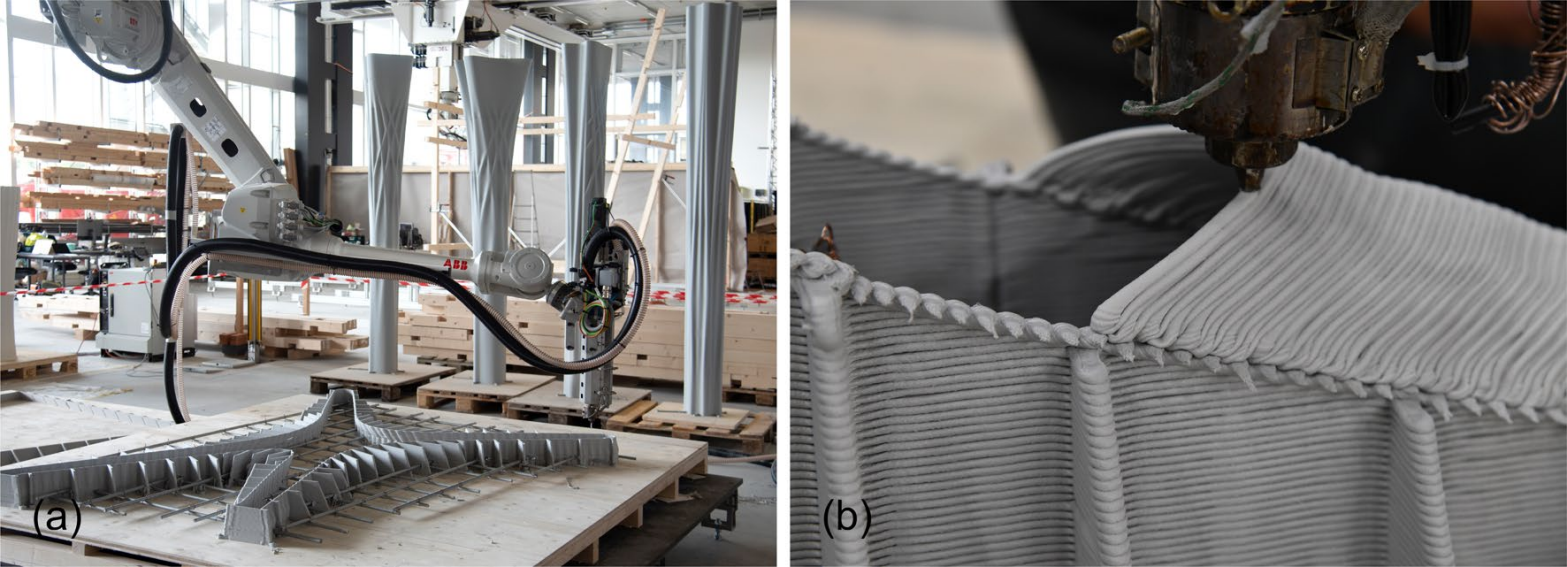
3D Printing process of a slab formwork. **a** 3D Printing the formwork walls, **b** 3D printing the cap

**Fig. 13:**
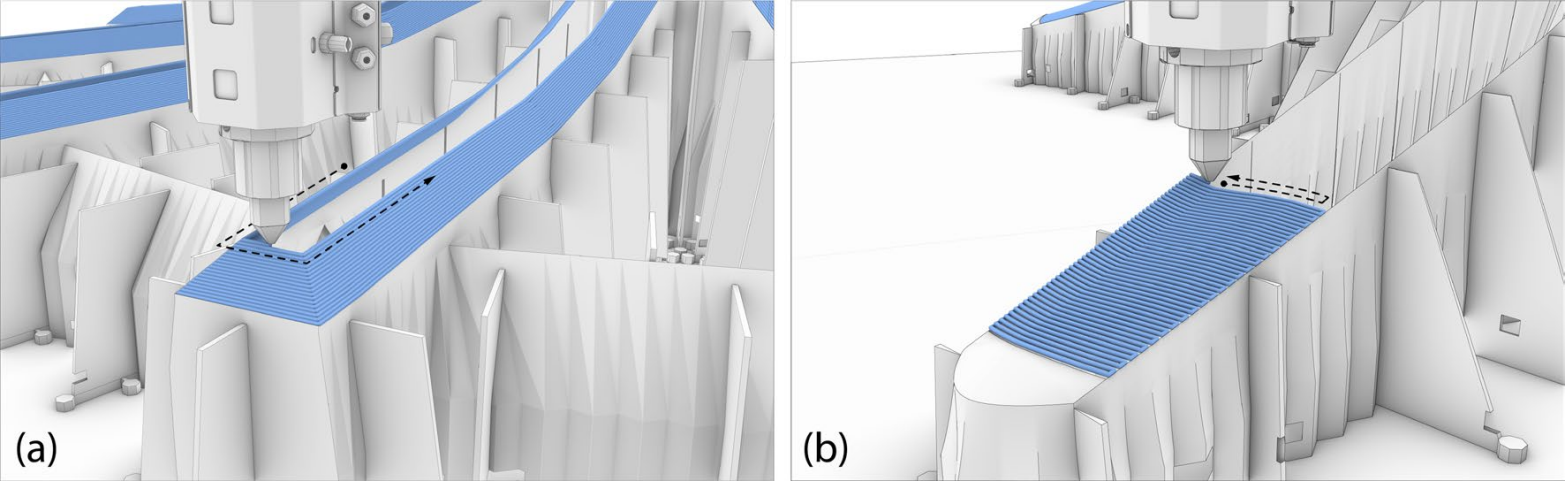
3D Printing of the caps. **a** 3D Printing toolpath for the caps of the RIBB3D project with a triangular cross-section (Burger et al. 2022), **b** 3D Printing toolpath for the caps of the Eggshell Pavilion, using a back-and-forth printing motion

The outer boundary and formwork walls were printed using planar layers, similar to the column formworks (Fig. 12a). However, it is impossible to print the cap using planar layers, as the below layers do not support the horizontal surfaces. A similar problem had been encountered in the RIBB3D project (Burger et al. 2022). In that case, this was solved by changing the flat cap to a pitched cap, which provided enough support for printing (Fig. 13a). However, this resulted in a cap with a triangular cross-section, which was not optimal from a structural perspective, as experiments had shown concrete spalling in the rib caps during structural testing (Huber et al. 2022).

For that reason, a different approach was attempted for 3D printing the cap. The cap geometry was designed to represent an arc in cross-section. As for the printing path, the cap of each rib would be printed separately (as opposed to all rib caps at the same time) in a back-and-forth printing motion (Figs. 12b, 13b).

The back-and-forth printing motion resulted in a printpath with a length of just 150 mm for each layer. Therefore, to achieve a time per layer of 15 s, it was necessary to reduce the printing speed to 10 mm/s. Additionally, as the print head moves back and forth between layers, it has to print the new layer on top of a supporting layer immediately after having finished printing the supporting layer. Due to these aspects, the printing speed had to be further reduced to 8 mm/s, despite having active cooling. This resulted in the caps having a similar printing time compared to the formwork walls, even though the formwork walls have a toolpath length that is around ten times greater than the caps.

Printing the caps proved challenging not only because of their long printing time but also because of the sagging of the print. Due to the short toolpath and insufficient cooling, the caps sagged during printing, which required occasional manual support during the process.

#### Reinforcement

The reinforcement bars for the solid slab part of the floor were inserted between the 3D printed layers in holes printed as part of the formwork (Fig. 11b). The reinforcement bars for the ribs were inserted as prefabricated cages (Fig. 11d, e).

In the RIBB3D study, it was challenging to place the reinforcing bars of the solid slab in the holes as there was not enough tolerance between the side of the holes and the bars. Therefore, the width of the holes was increased from 15 to 18 mm, which allowed for relatively easy insertion of the reinforcing bars.

The rib reinforcement cages were prefabricated by a concrete prefabrication company and inserted into the formwork. As the reinforcement had been designed with a concrete cover distance of only 25 mm, there was little space between the reinforcement cage and formwork. The lack of space made it challenging to insert the reinforcement cages, which made the reinforcement insertion process time-consuming, taking around 3 h per slab.

**Fig. 14:**
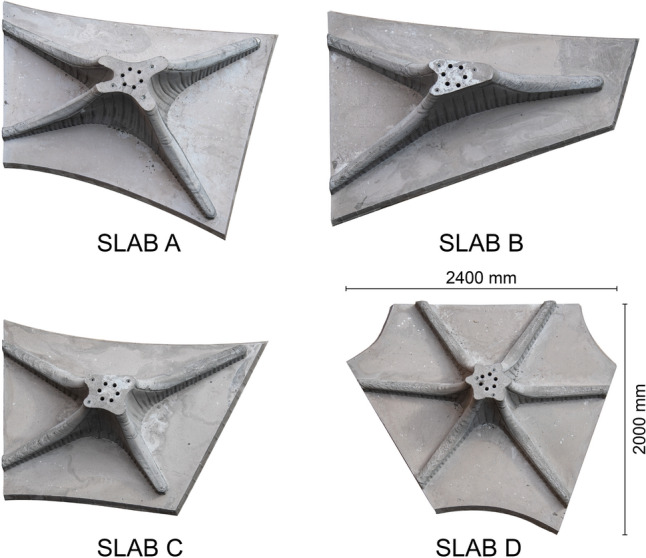
The four completed floor slab elements

#### Casting

After printing had been completed, the formworks were prepared for casting. Polyvinyl chloride (PVC) pipes were inserted into the formwork to create the voids that would connect the floor slabs to the columns. 3D Printed templates similar to the ones used for the columns (Sect. 6.1.3, Fig. 7) were used at the top and bottom to place the pipes in the correct position.

The floor slab formworks were cast using the self-compacting concrete described in Sect. 3.3. The reason for choosing a concrete recipe with a small aggregate size of 4 mm was to have a fluid mix that would easily flow everywhere into the formwork, despite only being cast from the centre of the formwork.

In contrast to the casting process of the columns (Sect. 6.1.3), the floor slabs were cast using conventional casting without accelerating admixtures. This meant that the formwork must be able to resist the hydrostatic pressure exerted on the formwork by the freshly cast concrete. Due to the different heights of the floor slab elements (Table 2), there was a different maximum hydrostatic pressure for each element. Slab A reached the highest pressure due to its large height (660 mm). During the casting of Slab A, the cap of one of the capital ribs disconnected from the formwork walls. This caused the rib to widen, resulting in concrete leakage between the formwork walls and the cap. The leakage was stopped using a clamp to stop the widening of the rib, which allowed the formwork to be filled entirely. The other three capitals could be cast without additional problems (Fig. 11g).

The capitals were demoulded two days after casting using a hot air gun and pliers. Demoulding of the elements was more time-consuming than demoulding of the columns, mainly because of three factors: (1) the formwork is slightly thicker (5 mm instead of 3 mm), (2) the stiffening ribs provide extra strength to the formwork, and (3) the reinforcing bars for the solid slab part of the formwork protrude through the formwork, making it difficult to remove.

After demoulding of the capital, the solid part of the slab could be cast (Fig. 11h), completing the fabrication process of the floor slabs (Fig. 14).

### Transport and assembly

**Fig. 15:**
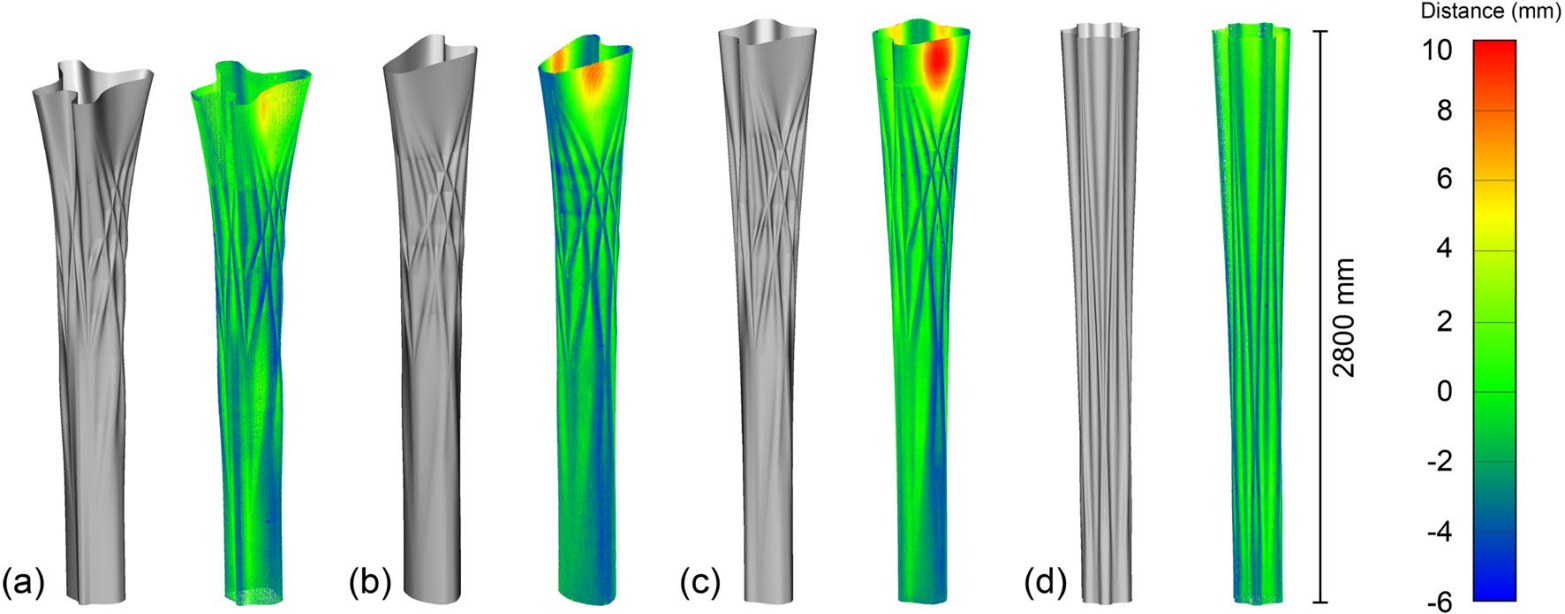
Comparison of the designed column geometry with the geometry obtained from 3D scanning. Left shows the as-designed mesh, right shows the scanned point cloud, colour coded to indicate the distance between the mesh and the point cloud. **a** Column A, **b** column B, **c** column C, **d** column D

**Fig. 16:**
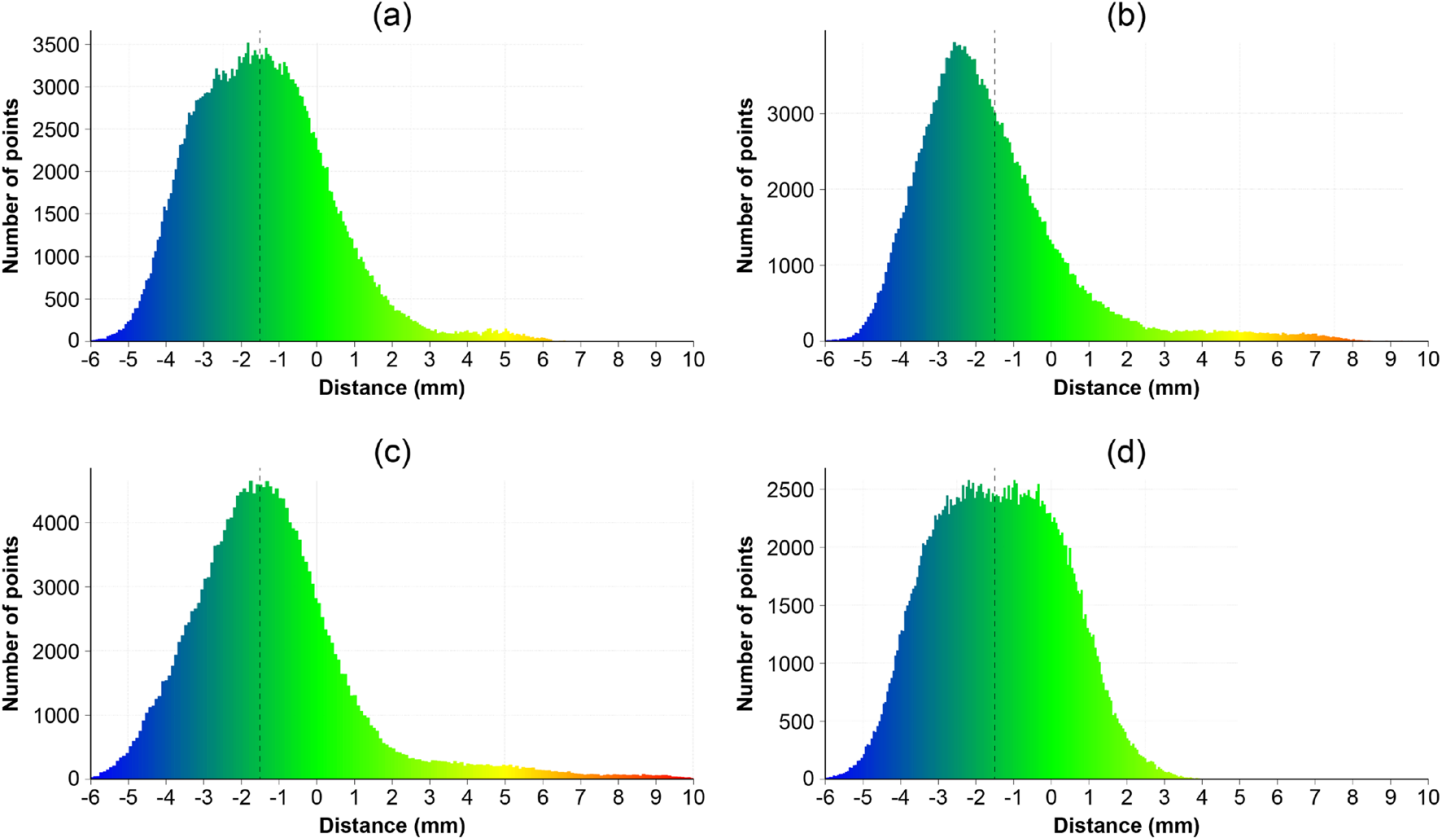
Column histograms. Representation of the distance between designed column geometry and scanned point cloud in a histogram. The *y*-axis indicates the number of points within a bar of the histogram, the *x*-axis indicates the distance. The dashed line at − 1.5 mm indicates what the optimum distance value would constitute. **a** Column A, **b** column B, **c** column C, **d** column D

After the concrete elements were finished, they could be transported to the construction site. The columns were fabricated vertically but needed to be laid horizontally for transport. This presented a challenge, as the non-standard shape of the columns required a custom solution. To avoid having to build a complex supporting frame, the columns were laid down, resting on the bottom steel plate and on a wooden plate that was screwed to the top of the column. Additionally, wooden beams were added between the two plates, as well as wooden supports to support the centre of the columns.

The floor slabs were transported on the wooden platforms used to print the formwork and cast the elements (as described in Sect. 6.2). As the floor slab elements were fabricated upside down, they had to be flipped 180$^{\circ }$ into their final position using a crane on the construction site.

On-site, the ground screw foundation was placed first, after which the steel connection plate could be placed and the columns connected to the base plate (Fig. 2). Then, the floor slab elements were placed on the columns and connected. The columns could be adjusted into their final position using the slotted holes in the base plates. Finally, the floor slabs were connected at the top using steel brackets.

### 3D scans

#### Columns

3D Scans of the finished columns were made using the method outlined in Sect. 3.4. The results can be seen in Table 3, Figs. 15, and 16. The standard deviation of the columns lies between 1.69–2.02 mm, which corresponds with the accuracy of the scanner (1.9 mm).

It is important to note that the scanned result was compared with the mesh used for 3D printing the formwork, which means that the thickness of the formwork (3 mm) has to be considered in the comparison. Therefore, if the scanned result is 1.5 mm smaller than the designed geometry, there is a perfect match between as-designed and as-fabricated. Generally, all columns have a mean distance relatively close to the optimum (− 1.5 mm). From the histograms (Fig. 16) can be seen that most of the points are centred around the optimum (− 1.5 mm). Column C has the highest deviation from the optimum (+ 0.23 mm), most likely due to the large deviations at the top of the column.

Columns B and C have the highest positive deformation, meaning the finished object is larger than the designed geometry. Figure 15 shows that this is due to large deformation at the top of the formwork. Both Columns B and C have long straight lines vulnerable to bending due to the hydrostatic pressure from the concrete (Burger et al. 2021). However, Column D only shows minor deformation at the top due to its undulating shape.

**Fig. 17:**
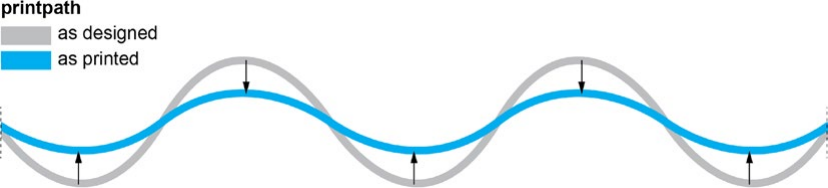
Illustration of a top view of a printed layer, showing the dragging of filament towards the inside when printing an undulated printpath. Grey shows the path as it was designed, and blue shows the path as it is actually printed, resulting in lower peaks and shallower valleys

All columns have a relatively similar maximum negative distance (− 5.54 to − 8.84), meaning the finished object is smaller than the designed geometry. Most of the places where the columns are smaller than intended are due to casting defects, as seen in Fig. 9a. However, some deviation can also be observed in the pattern of the columns. In particular, the peaks of the pattern are smaller than designed, whereas the valleys are larger. Most likely, this is the result of dragging the printed filament towards the inside during 3D printing of an undulating path, resulting in lower peaks and shallower valleys (Fig. 17).

**Table 3:** Results of the 3D scanning process of the columns

	Column A	Column B	Column C	Column D
Max positive distance (mm)	6.62	8.75	10.51	4.21
Max negative distance (mm)	− 6.78	− 6.30	− 8.84	− 5.54
Mean distance (mm)	− 1.44	− 1.69	− 1.27	− 1.40
Point density ($\text{pts/cm}^{2}$)	8.58	8.28	8.46	8.38
Standard deviation (mm)	1.78	1.90	2.02	1.69

A positive distance means that the scanned point cloud is larger than the designed geometry, negative distance means the scanned point cloud is smaller than the designed geometry

#### Floor slabs

**Fig. 18:**
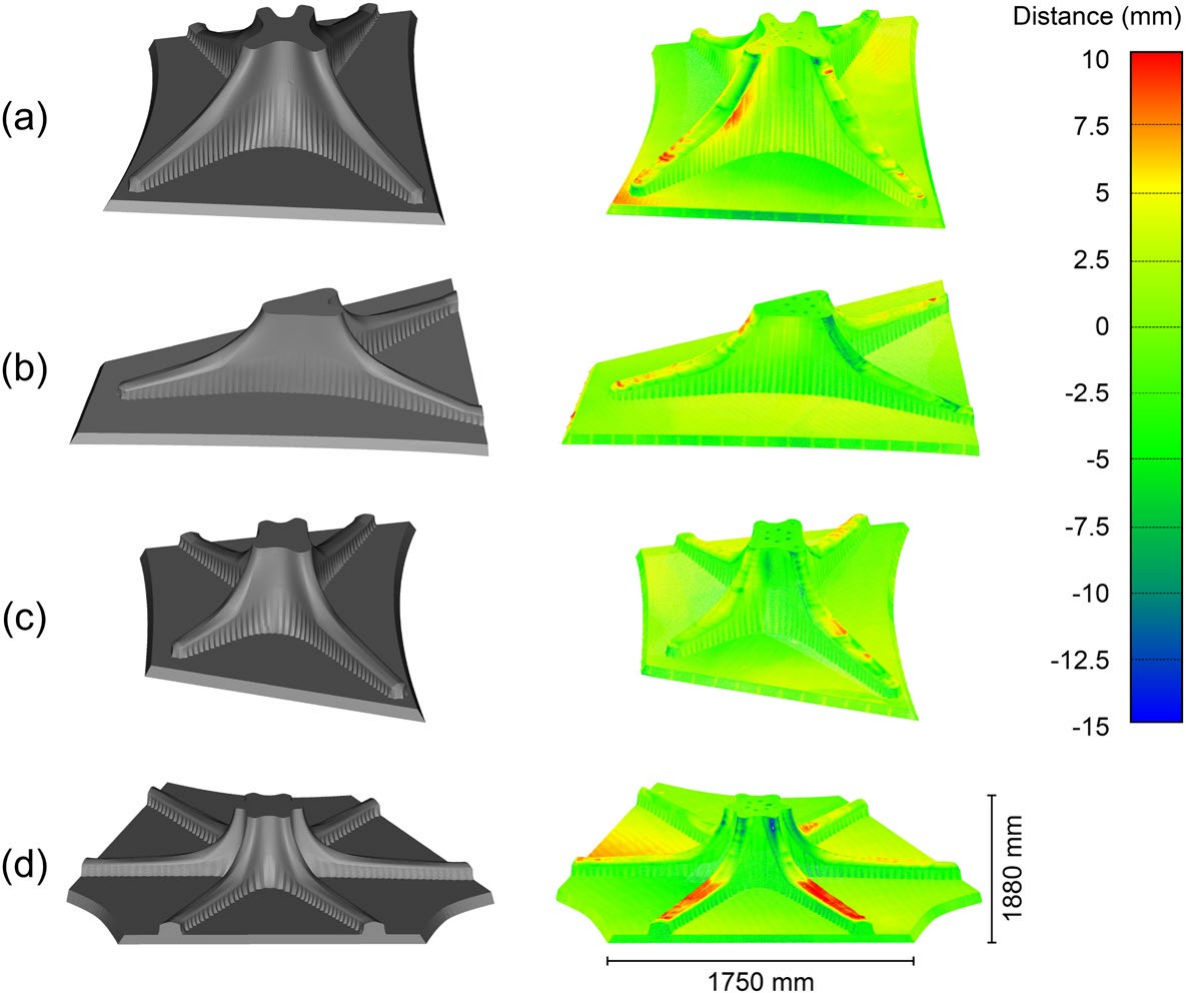
Comparison of the designed floor slab geometry with the geometry obtained from 3D scanning. Left shows the as-designed mesh, right shows the scanned point cloud, colour coded to indicate the distance between the mesh and the point cloud. **a** Slab A, **b** slab B, **c** slab C, **d** slab D

3D Scans of the finished floor slab elements were made using the method outlined in Sect. 3.4. The results can be seen in Table 4 and Fig. 18. Similar to the columns, the thickness of the formwork (5 mm) still has to be considered in the results, meaning a perfect match between the designed and fabricated geometry would be a distance of − 2.5 mm. Generally, all the floor slabs are between 2.13–1.5 mm larger than they should be.

This inaccuracy is mainly caused by the caps, most of which are larger than intended. In particular, caps with more horizontal surfaces are much larger, such as for slab D (Fig. 18d). Most likely, this is the result of manual intervention, as the caps had to be manually supported during the printing process.

Moreover, the upper part of the caps (towards the transition to the column) is typically smaller than the designed geometry. One reason for this inaccuracy could be that the extruder was not oriented vertically during the printing of that section. Instead, the nozzle pointed inwards, which we believe resulted in the formwork being printed smaller than designed.

The vertical formwork walls are more accurate, with the largest part of the fabricated element being within a distance of 2.5 mm (− 5 to 0 mm) from the intended design.

**Table 4:** Results of the 3D scanning process of the floor slabs

	Slab A	Slab B	Slab C	Slab D
Max positive distance (mm)	12.16	9.42	8.10	12.62
Max negative distance (mm)	− 13.28	− 14.54	− 11.48	− 14.77
Mean distance (mm)	− 0.37	− 1.00	− 0.91	− 0.65
Point density ($\text{pts}/\text{cm}^{2}$)	10.39	9.88	10.37	10.99
Standard deviation (mm)	2.72	2.65	2.63	3.35

A positive distance means that the scanned point cloud is larger than the designed geometry, negative distance means the scanned point cloud is smaller than the designed geometry

## Discussion

**Fig. 19:**
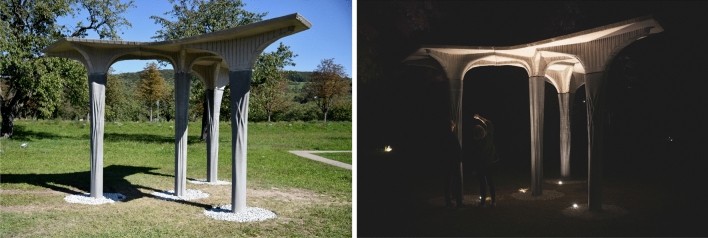
The completed pavilion (Image: Joris Burger, Jingwen Wang)

The fabrication of the Eggshell Pavilion shows the potential of using 3D printed formwork to build a non-standard structure consisting of columns and slabs (Fig. 19). This illustrates a number of potential advantages but also limitations for construction.

### Advantages

Employing a digital design-to-fabrication process, such as described in this paper, has several advantages. As the pavilion geometry was completely parametrically modelled, the design could easily be modified by adjusting the parameters of the design workflow (as described in Sect. 5.1). Since the entire process, from the generation of the pavilion outline to the precise formwork geometry, was automated, changes to the geometry did not require any manual remodelling but were updated automatically. The digital model also allowed for easy data exchange with the structural engineer without having to produce additional drawings. Moreover, the drawings for producing reinforcement bars could be automatically generated from the digital model, and no drawings needed to be created for the production of the formwork. Lastly, fabrication constraints (such as printing time) could easily be taken into account during the conceptual design of the project.

The use of 3D printing to produce the formwork for the columns and floor slab elements allowed for geometrical freedom whilst automating the fabrication process. The column formworks, in particular, were 3D printed in around seven hours without any manual intervention. This increased geometrical freedom could allow for producing non-standard, material-optimised concrete elements. Here lies a strong potential for more sustainable construction, especially in floor slabs, where material savings of 40–70% can be achieved (Burger et al. 2022; Ranaudo et al. 2011; Meibodi et al. 2018).

In the design, fabrication, and construction process of the Eggshell Pavilion novel, digital methods were combined with conventional techniques. The pavilion was designed using algorithms, and the formwork was fabricated using robotic arms, but the reinforcement was assembled manually, as were the building elements on the construction site. This combination shows that novel design and fabrication methods, such as parametric design and 3D printing, can be compatible with existing construction methods, such as manual assembly of reinforcement.

The successful fabrication of the four columns shows the potential of using fully recycled material to 3D print formworks. Although the cycle of 3D printing, formwork removal, shredding, and reprinting has only been completed once, it shows how the formwork used to produce a non-standard element can directly be recycled to produce new formwork for another bespoke element.

Lastly, 3D printed formwork allows for adding ornament to concrete structures without additional cost. For example, the printing time of the formwork for the columns was dictated by the minimum time per layer, not by the maximum printing speed. This means that a layer with a longer toolpath can be printed at a higher printing speed to achieve the same time per layer. Therefore, a slightly longer printpath per layer, due to an ornament, does not increase printing time.

### Limitations and possible improvements

Although the presented design-to-fabrication workflow is well-suited for the design and production of the Eggshell Pavilion, it would need to be adapted to be applied to different projects. In particular, the first step of the computational workflow, in which the overall outline is defined, would require modification to accommodate for additional columns and floor slab elements.

The 3D printing process of the floor slab formworks presented some issues. In particular, the printing process of the caps was very slow due to the low speed required to ensure proper cooling (Sect. 6.2.1). After casting, the caps showed high inaccuracies compared to the rest of the model (Sect. 6.4.2). Moreover, one of the caps detached from the formwork walls during casting due to a weak bond between the formwork walls and the cap. Therefore, the printing process of the caps will have to be rethought in future work.

Several aspects of the digital casting process of the columns still need improvement. The process must become more robust to function reliably under changing ambient conditions and when casting over longer time periods. This could help to avoid defects on the column surfaces (Fig. 9a). Additionally, the concrete recipe has to become more sustainable: less cement and a higher percentage of larger aggregates (Boscaro et al. 2022; da Silva et al. 2022; Flatt and Wangler 2022). Lastly, to industrialise the process, the flow rate of the digital casting process needs to be increased so that the columns can be cast faster (Lloret-Fritschi et al. 2022).

The columns were fabricated with relatively high precision, with most sections within the construction tolerance of five millimetres (Sect. 6.4.1). However, the top section of the columns showed inaccuracies of up to 12 mm. The top accuracy of the columns is even more crucial given that the column top connects to the floor slab elements. Potentially, accuracy could be improved by confining the top geometry using a laser-cut template. Additionally, shapes can be designed that are less sensitive to deformation due to hydrostatic pressure, i.e., convex instead of concave cross-sections (Burger et al. 2021).

Despite the automated fabrication of the formwork for the columns and floor slabs, there is still a high amount of manual labour necessary. Generally, the number of hours spent on manual labour exceeds machine hours by a large margin. For the columns, a large amount of manual labour was spent on material preparation and casting. Currently, the material is prepared in a laboratory facility. If instead, the material is prepared in an industrial facility, this would already be improved.

For the floor slabs, most manual labour was spent on demoulding the formwork, as well as the placement of the reinforcement. These processes are difficult to automate; however, for the proposed fabrication process to be applied at an industrial scale, it is crucial that the demoulding process and the reinforcing process are improved. Further strategies for this are discussed in Burger et al. (2022).

Generally, the cost and time spent on manual labour far exceed the cost and time spent on the 3D printing process. Therefore, despite the formwork production process being more optimised, this made other processes (such as reinforcement, or demoulding) more laborious and cost-intensive. In particular, the demoulding process of the slabs was time-consuming. A more elaborate cost and time analysis, such as that conducted by Soto et al. (2018) will have to be done to make a proper comparison.

Furthermore, the additional time spent preparing the transport elements can also not be neglected. As each element is unique, each requires different packaging, which increases cost. To scale up the prefabrication process of non-standard concrete elements, a method will have to be found to transport the elements in an efficient way. Additionally, the possibility of casting concrete on-site, possibly in a movable shelter, could be explored. This may reduce transport issues, although it increases sensitivity to temperature variations.

Lastly, due to the chosen constructive system of auto-stable elements and the limited fabrication size, the pavilion spans were limited to around two meters. For the column-slab system to be applied in an actual construction project, larger spans (5–8 m) will need to be realised. This would require the development of more sophisticated slab-slab connections that can transfer compressive, shear, and tensile stresses between adjacent elements (Bischof et al. 2022).

## Conclusion and outlook

This paper describes the design, fabrication, and assembly process of the Eggshell Pavilion, a full-scale reinforced concrete structure fabricated using robotically 3D printed recycled formwork. The successful fabrication of the pavilion shows that the presented process is a viable method of building non-standard concrete structures. The formwork can be produced in an automated process, reducing the need for labour-intensive, manual formwork production methods. The digital design-to-fabrication process reduces the need to produce drawings and allows implementing changes during the process with relative ease. Due to the compatibility with existing construction practices, it is already possible to use the process on a construction site.

Despite the benefits, there are still challenges that need to be addressed before the process can reach mass-market adoption, such as increasing process robustness, increasing automation, and improving sustainability. By enabling the fabrication of non-standard concrete structures, it could be possible to reduce the amount of concrete needed for structural building components. It is crucial for this technology to make a positive impact, that these improvements in material efficiency will not be offset by aspects such as unsustainable concrete mixes and transportation complexity. Economical and environmental analysis would need to be conducted in future work to study the added benefits of material efficiency versus the extra impact of the production process. However, the used materials can be improved, and specific improvements can be made to the fabrication process. Then, digital design and fabrication processes such as those presented in this paper can aid the transition toward more sustainable construction with concrete.

## Supplementary Information

Below is the link to the electronic supplementary material.

Supplementary file1 (MP4 298032 KB)

## Data Availability

The datasets generated during and/or analysed during the current study are available from the corresponding author on reasonable request.
